# Natural Killer Cell Signal Integration Balances Synapse Symmetry and Migration

**DOI:** 10.1371/journal.pbio.1000159

**Published:** 2009-07-28

**Authors:** Fiona J. Culley, Matthew Johnson, J. Henry Evans, Sunil Kumar, Rupert Crilly, Juan Casasbuenas, Tim Schnyder, Maryam Mehrabi, Mahendra P. Deonarain, Dmitry S. Ushakov, Veronique Braud, Günter Roth, Roland Brock, Karsten Köhler, Daniel M. Davis

**Affiliations:** 1 Division of Cell and Molecular Biology, Imperial College London, London, United Kingdom; 2 National Heart and Lung Institute, Imperial College London, London, United Kingdom; 3 Institut de Pharmacologie Moleculaire et Cellulaire, Centre National de la Recherche Scientifique/Université de Nice-Sophia Antipolis, UMR6097, Valbonne, France; 4 Department of Molecular Biology, Interfacultary Institute for Cell Biology, University of Tübingen, Tübingen, Germany; 5 Department of Biochemistry, Nijmegen Centre for Molecular Life Sciences, Radboud University Nijmegen Medical Centre, Nijmegen, The Netherlands; National Jewish Medical and Research Center/Howard Hughes Medical Institute, United States of America

## Abstract

Natural killer (NK) cells discern the health of other cells by recognising the balance of activating and inhibitory ligands expressed by each target cell. However, how the integration of activating and inhibitory signals relates to formation of the NK cell immune synapse remains a central question in our understanding of NK cell recognition. Here we report that ligation of LFA-1 on NK cells induced asymmetrical cell spreading and migration. In contrast, ligation of the activating receptor NKG2D induced symmetrical spreading of ruffled lamellipodia encompassing a dynamic ring of f-actin, concurrent with polarization towards a target cell and a “stop” signal. Ligation of both LFA-1 and NKG2D together resulted in symmetrical spreading but co-ligation of inhibitory receptors reverted NK cells to an asymmetrical migratory configuration leading to inhibitory synapses being smaller and more rapidly disassembled. Using micropatterned activating and inhibitory ligands, signals were found to be continuously and locally integrated during spreading. Together, these data demonstrate that NK cells spread to form large, stable, symmetrical synapses if activating signals dominate, whereas asymmetrical migratory “kinapses” are favoured if inhibitory signals dominate. This clarifies how the integration of activating and inhibitory receptor signals is translated to an appropriate NK cell response.

## Abstract 2

Imaging immune surveillance by natural killer (NK) cells has revealed that integration of activating and inhibitory signals determines whether or not NK cells stop to kill the target cell or retain a migratory configuration.

## Author Summary

Immune cells survey their local environment and an immunological response can be activated when an appropriate target cell or antigen-presenting cell is recognised by key cell surface molecules. Just how the multitude of protein–protein interactions work to regulate this decision is an ongoing question. Imaging technology has provided key insights, demonstrating that immune cell activation is often accompanied by the segregation of proteins at immune synapses. Natural killer (NK) cells are lymphocytes that can recognise and kill virally infected or tumour-transformed cells via the formation of a synapse that facilitates secretion of cytotoxic granules directed at the target cells. Key to understanding target cell recognition by NK cells is to establish how the balance of activating and inhibitory signals at the synapse leads to an appropriate response, e.g., to kill or spare a target cell. We found that when activating ligands are dominant on a target cell, NK cells stop migrating and spread lamellipodia across the target cell to form a large symmetrical synapse. If inhibitory signals dominate, the symmetry of the NK cell spreading response is broken and the stop signal is reversed, which reduces the time spent in contact with the target cell. Thus, NK cell activating and inhibitory signals regulate NK cell synapse symmetry and migration to determine whether an NK cell will kill or move on.

## Introduction

Natural killer (NK) cells are lymphocytes that play an important role in defence against infections and cancer [1]. They directly kill tumor-transformed or virally infected cells via the formation of a cytolytic synapse that facilitates polarisation and subsequent secretion of cytotoxic granules directed towards the target cell [2]–[4] and also contribute to immunity through, for example, cytokine release [5]. NK cell activation is regulated by a balance of activating and inhibitory signals through a multitude of germ-line encoded receptors that recognise ligands expressed on the surface of other cells [6],[7]. One way that cells become susceptible to NK cell lysis is through an upregulation of activating ligands at their surface. One of the best characterised activating receptors on NK cells, NKG2D, recognises “stress-inducible” ligands, such as MICA, which are upregulated following, for example, heat shock or UV-induced DNA damage [8],[9]. Alternatively, loss of inhibitory ligands from the surface can also render cells susceptible to NK lysis. Inhibitory receptors include the killer-cell immunoglobulin-like receptors (KIRs), members of the leukocyte Ig-like receptor (LILR) family, and the CD94:NKG2A heterodimer that recognise class I MHC molecules [10],[11]. Broadly, activating receptors trigger signalling cascades via phosphorylation of ITAM or ITAM-like motifs encoded within activating receptors or, more commonly, within associated adaptor proteins, whereas inhibitory receptors contain ITIM motifs that recruit SH2 domain protein tyrosine phosphatases upon receptor engagement (recently reviewed in [6]).

Understanding how the balance of activating and inhibitory signals in NK cells leads to appropriate effector responses is an important challenge in NK cell biology [12]. NK cells form synapses with target cells in which cell surface molecules accumulate and organise into micrometer-scale domains [2],[3],[13]. At lytic synapses, f-actin accumulates at the synapse, the microtubule organizing centre (MTOC) polarises, and lytic granules are directed towards the target cell [2],[14]–[16]. However, it remains a central gap in our knowledge as to how the balance of activating and inhibitory signals relates to these dynamic cell behaviours and controls the eventual outcome of the interaction [17].

A special characteristic of our immune system compared with, for example, the nervous system is the high motility of immune cells and their propensity to form dynamic intercellular conjugates, or so-called “make and break” synapses [18],[19]. Moreover, recent imaging of immune cell interactions in vivo found that a complex orchestration of cell–cell contact times is a key correlate to establishing appropriate immune responses. Integration of competing receptor signals for migration or adhesion likely determine the duration of cell–cell contacts, and thus understanding the regulation of immune cell motility is important for how signal integration leads to appropriate effector functions in these cells. In homeostasis, T cells scan for specific peptides presented by dendritic cells by moving rapidly in lymph nodes [20],[21]. When a cognate antigen is encountered, T cells stop and form a prolonged interaction with the antigen presenting cells [22]–[25]. Migrating cells are polarised and asymmetrical, consisting of a leading lamellipodium, a lamella, and trailing uropod [26], whereas on activation T cells spread symmetrically to form a stable synapse [27],[28] to which peptide/MHC proteins are recruited and signalling is sustained within microclusters of proteins that assemble at the synapse periphery [29],[30]. A migratory configuration of T cells, which allows moving contacts with antigen presenting cells, has recently been termed a “kinapse” [27],[31].

Here, we demonstrate that ligation of the cell surface integrin LFA-1 alone on NK cells can trigger asymmetrical spreading to aid cell migration. In contrast, ligation of activating receptor NKG2D imposes symmetrical cell spreading via extension of sheets of lamellipodia containing a ring of f-actin. NKG2D ligation also delivers a stop signal to halt NK cell migration. Importantly, inhibitory signalling is integrated continuously with activating signals during spreading and serves to block symmetrical spreading and provides a reverse-stop signal. The size of inhibitory synapses and the dwell time of NK cells on inhibitory targets are consequently reduced. Thus, our data suggest that in NK cells, a dominance of activating signals results in symmetrical spreading to form a large stable synapse, whereas a dominance of inhibitory signals results in a smaller less stable migratory “kinapse” thereby preventing cytotoxicity.

## Results

### NK Cells Undergo a Spreading and Contraction Response at Activating but Not Inhibitory Synapses

**Figure 1:**
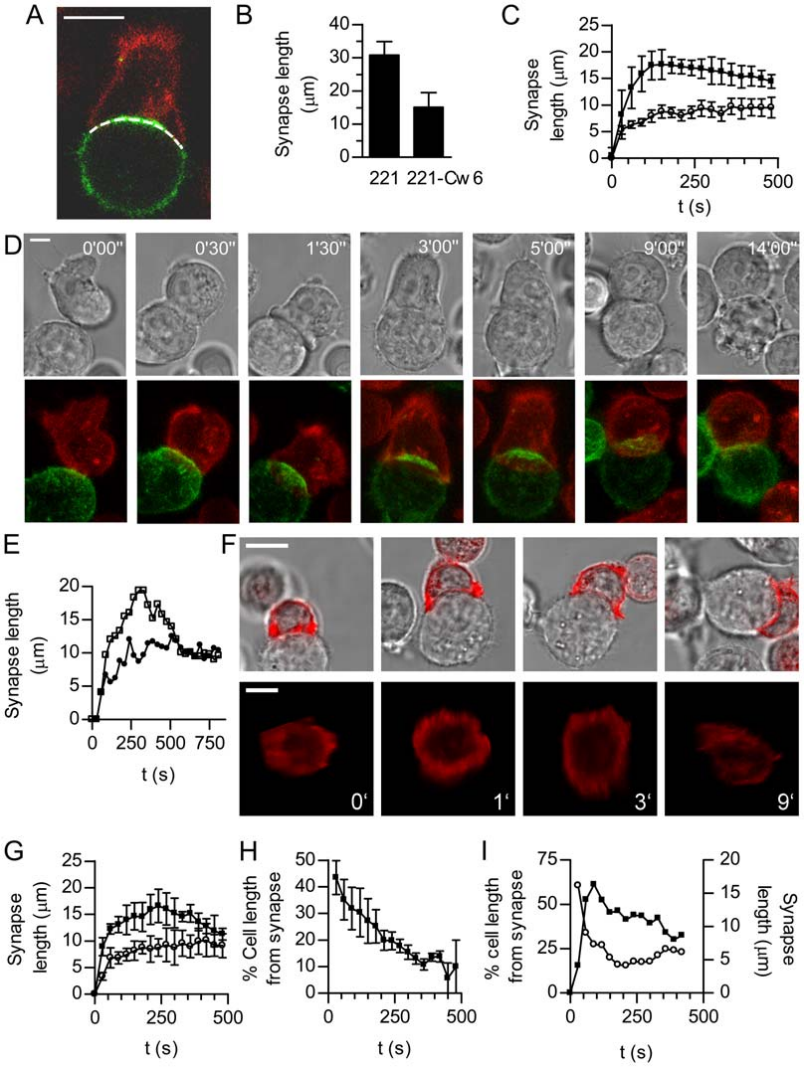
The NK cell spreading and contraction response. (A) An illustrative optical slice through a conjugate between a DiO-labelled NK cell (red) and a transfectant of 221 expressing MICA-YFP (green). The dotted line illustrates the length of contact measured. Scale bar  = 10 µm. (B) The synapse length (mean + SD) at activating synapses between YTS/KIR2DL1 and 221 target cells (*n* = 6) or inhibitory synapses between YTS/KIR2DL1 and 221/Cw6 (*n* = 13). (C) The size of activating synapses (mean±SD) between YTS/KIR2DL1 and 221 (*n* = 9, squares), and inhibitory synapses between YTS/KIR2DL1 and 221/Cw6 (*n* = 7, circles). (D) Time-lapse microscopy of NKL (red) spreading and contracting on a 221 cell expressing MICA-YFP (green). Images shown are maximal projections of fluorescent images and corresponding bright field images. Time as indicated, scale bar  =  5 µm. (E) The synapse length for the cell shown in (D) (squares) and a representative inhibitory synapse between an NKL cell and a 221-HLA-E (circles). (F) Time-lapse microscopy of NKL expressing actin-YFP and a 221 cell is shown as bright field images, overlaid with YFP-actin (red) and as an en face view of actin-YFP at the synapse. Times indicated relative to first image, scale bars  =  10 µm. (G) The length of synapse (mean±SD) for NKL cells and 221 expressing MICA-YFP (activating synapse; *n* = 5, squares) or 221 expressing HLA-E (inhibitory synapse; *n* = 7, circles). (H) Polarisation in NKL cells with 221-MICA, expressed as the distance of the 3-D centroid of intracellular membrane vesicles stained with DiD from the centre of the synapse relative to the length of the cell (mean±SEM; *n* = 7). (I) Measurement of synapse length (solid squares) reveals the spreading and contraction between a primary NK cell and 221 target cell. The distance of intracellular membranes from the synapse (open circles) highlights the simultaneous cell polarisation towards the target cell. Data are from a single conjugate, representative of 15.

Broadly, NK cells may form at least two types or categories of synapse; an activating synapse that triggers target cell killing (and other effector functions), or an inhibitory synapse, in which signals from inhibitory ligands dominate and the target cell is spared (and other effector functions are blocked). Here, we first set out to compare the sizes of inhibitory and activating synapses. Confocal images of NK cell–target cell conjugates were acquired and the maximum length of the cell contact in a single *x*-*y* plane was measured (Figure 1A). To readily compare activating and inhibitory NK cell synapses, we initially used the well-established model cell system of the NK cell line YTS transfected to express an inhibitory receptor KIR2DL1 (YTS/KIR2DL1) mixed with either untransfected 721.221 (221), which does not express endogenous HLA-A, -B, or -C, or transfectants of 221 (221/Cw6) expressing an inhibitory MHC ligand for KIR2DL1, HLA-Cw6. This system is well-established as functionally leading to NK cell activation or inhibition [13]. The synaptic contact was 30.8±4.2 µm between YTS/KIR2DL1 and 221, i.e., at an activating synapse, compared to 15.1±4.4 µm between YTS/KIR2DL1 and 221/Cw6, i.e., an inhibitory synapse. Thus there is a 4-fold increase in contact area at activating synapses relative to inhibitory contacts in this system (Figure 1B).

To determine the kinetics of these morphological changes at activating and inhibitory synapses, the diameter of the contact between cells was measured every 30 s. For YTS cells interacting with 221 cells, i.e., at activating synapses, NK cells rapidly spread over the surface of the target cell, reaching their maximum area on average 2 min after contact, which was followed by a contraction (Figure 1C). In contrast, when HLA-Cw6 was expressed by target cells, the inhibitory synapse expanded less rapidly and remained small (Figure 1C).

To test the generality of these observations, another NK cell line, NKL, was mixed with transfectants of 221 expressing MICA-yellow fluorescent protein (YFP), a ligand for the activating receptor NKG2D expressed by NKL. As for YTS, NKL exhibited a spreading and contraction response at activating contacts that followed a similar time course (specific example shown in Figure 1D and 1E). Accumulation of MICA-YFP occurred during the spreading process, implying that contraction was not necessary to accumulate ligand at the synapse. Images of the en face synapse, in transfectants of NKL expressing YFP-actin, revealed that a ring of actin rapidly accumulated at the periphery of the activating synapse (Figure 1F). This ring persisted throughout the spreading phase and dissipated after the contraction phase.

HLA-E delivers an inhibitory signal to NKL cells via the CD94:NKG2A heterodimeric receptor. We confirmed that HLA-E expressed on 221 cells was able to inhibit NKL function, as interferon gamma (IFNγ) production by NKL was inhibited for target cells expressing HLA-E (Figure S1), consistent with previous studies [32]. More importantly here, recognition of the inhibitory ligand HLA-E, expressed in transfectants of 221, also prevented the spreading and contraction response in NKL (Figure 1E and 1G). To determine how spreading at the activating synapse is temporally related to the polarisation of intracellular granules and vesicles, trafficking of intracellular membranes was monitored in cells labelled with the membrane dye DiD. Polarisation, i.e., the distance of the centroid of the intracellular membranes to the synapse centre as a proportion of the cell length, began immediately after cell contact and continued concurrently with spreading (Figure 1H). Similarly, primary peripheral blood NK cells underwent a spreading and contraction response on target 221 cells, and this also coincided with the polarisation of granules towards the target cell (Figure 1I). Taken together, these data extend observations of NK cell flattening induced by activating ligands made by others [33] and establish temporal differences in the morphology of the inhibitory and activating NK synapse; interaction with an activating target cell triggers NK cells to spread to form a large synapse concurrent with formation of an actin ring and polarisation of intracellular granules, which is followed by a contraction phase and dissipation of the actin ring. In contrast, during inhibitory interactions the size of the contact between the NK cell and the target cell remains small.

### NKG2D and LFA-1 Ligation Trigger Morphologically Different Spreading Responses in NK Cells

**Figure 2:**
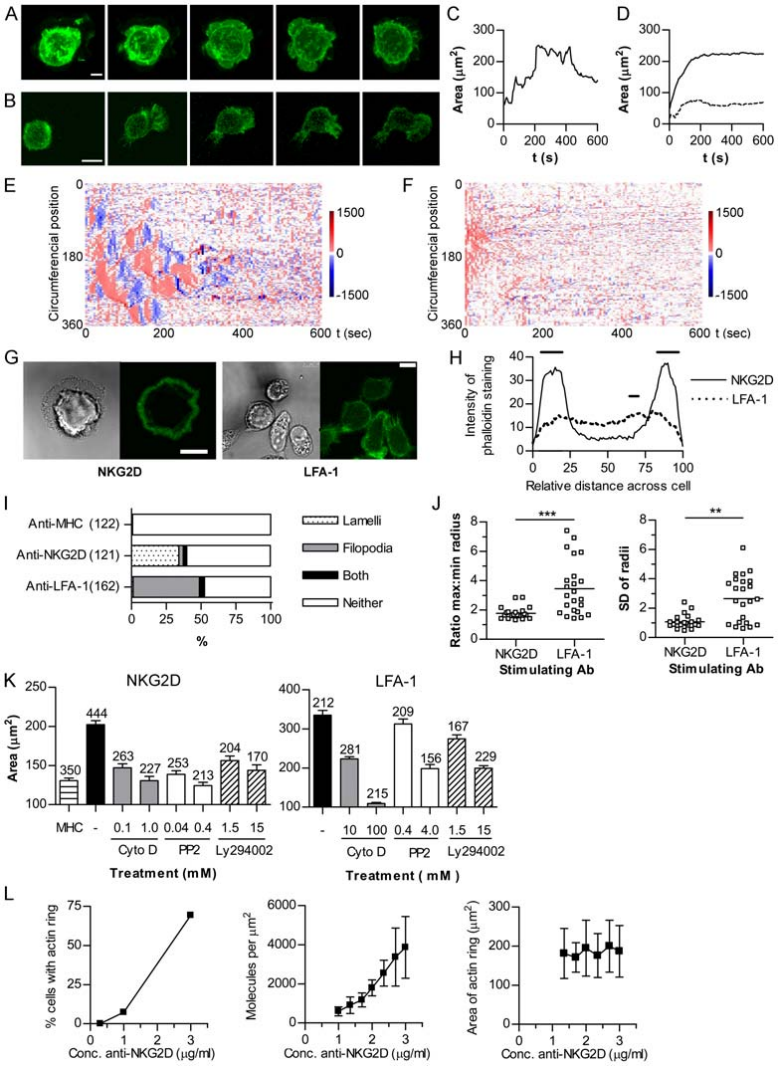
NKG2D and LFA-1 ligation trigger morphologically different responses in NK cells. Maximal intensity projections of YFP-mem NKL on (A) anti-NKG2D (scale  =  10 µm) or (B) anti-LFA-1 coated slides (scale  =  5 µm). Stacks (left to right) taken every minute from first contact with the slide. (C) Contact area of a single representative NKL cell spreading and contracting on anti-NKG2D, determined by confocal microscopy (images taken every 5 s). (D) Area of spreading of NKL cells on anti-LFA-1, untreated (solid line) or treated with sodium azide (dotted line). (E) The rate (nm/s) of extension or contraction of a YFP-mem NKL cell on anti-NKG2D, determined every 5 s. Extension away from the cell centre is coloured red and contraction in blue for each point around the circumference (*y*-axis) against time (*x*-axis). (F) Rate of extension or contraction of an NKL cell on anti-LFA-1. (G) F-actin (phalloidin-488; green), with corresponding bright field images for NKL cells on anti-NKG2D- or anti-LFA-1-coated slides. Scale  =  5 µm. (H) Quantification of f-actin (phalloidin-AlexaFluor633) across the diameter of cells stimulated on anti-NKG2D (solid line) or anti-LFA-1 (dotted line) (horizontal bars indicate *p*<0.05 by ANOVA, *n* = 20). (I) Morphology of cells stimulated with anti-LFA-1, anti-NKG2D, or anti-MHC I. Number of cells in brackets. (J) The distance from the centroid of phalloidin-AlexaFluor633-stained cells to the circumference was measured at 360 radii. Cell symmetry is represented as SD of the radial distances and the ratio of maximum and minimum lengths (**, *p*<0.01; ***, *p*<0.001; *n* = 20). (K) Spreading area (mean±SEM, *n* above each bar) of cells pretreated with cytochalasin, PP2, or LY294002, on anti-NKG2D, anti-LFA-1, or control untreated cells on anti-MHC I. (L) The density of antibody on the slide (*n* = 4±SD), the proportion of NKL cells responding by the formation of an actin ring and the area of spreading of cells forming an actin ring (mean±SD; *n* = 120–250) at a range of concentrations of anti-NKG2D.

To identity the minimal requirements for a spreading response in NK cells, glass slides were coated with mAb against NKG2D, a major activating receptor on NKL. NKL transfected to express membrane-bound (palmitoylated) YFP (YFP-mem), or stained with the membrane dye DiO, were observed to be activated by the mAb-coated surface and rapidly extended sheets of lamellipodia out across the surface of the slide, which was followed by a contraction phase during which the lamellipodia retracted (maximum projections; Figure 2A; Video S1). NKL did not undergo a spreading response on control isotype-matched or anti-MHC I mAb-coated slides, and the response to immobilised anti-NKG2D could be blocked by the addition of excess anti-NKG2D mAb to the culture supernatant (unpublished data). Furthermore, NKL used in this study did not express the Fc receptor CD16 (determined by flow cytometry; unpublished data) [34]. Thus, the NK cell spreading and contraction response can be triggered by ligation of NKG2D alone.

Ligation of LFA-1 induced a morphologically distinct response to that triggered by NKG2D (Figure 2B). Cells extended asymmetrically into an elongated, rather than round, contact area with the slide. Bright-field, fluorescence, and interference reflection microscopy (IRM) images were taken at the interface of cells with the surface of the glass slide, and the extent of spreading determined by quantifying the contact area. In response to NKG2D ligation, spreading was followed by a slower contraction response (Figure 2C). Analysis of the rate of change of the contact area of an average of 18 cells responding to NKG2D ligation, revealed a rapid initial spreading rate of 2.3±0.9 µm^2^/s triggered on contact with the surface, becoming negative (i.e., contracting) at 6 min after contact with the slide (average rate of contraction from 6 to 12 min after contact was −0.16±0.1 µm^2^/s). In contrast, spreading triggered by LFA-1 ligation resulted in a rapid spreading that was not followed by a contraction phase (Figure 2D). The response to LFA-1 was inhibited by the addition of sodium azide, confirming this was an active ATP-dependent process and not merely adhesive (Figure 2D).

We next analysed the direction of expansion of NK cells following NKG2D ligation by measuring the rate of change of the distance from the initial centre of the cell contact to the cell perimeter, around 360 equally spaced radii [27]. This analysis revealed that spreading in response to NKG2D occurred in waves which extended in all directions around the cell periphery, followed by a more quiescent phase during which the cell slowly contracted (Figure 2E). In contrast, analysis of the response to LFA-1 ligation revealed that cells spread rapidly and asymmetrically (Figure 2F). In response to NKG2D, but not LFA-1, a ring of f-actin was formed at the periphery of the area of contact of the NK cell with the slide, corresponding with the periphery of the extending lamellipodia (Figure 2G and 2H). Cells spreading on anti-LFA-1 did not form a ring of f-actin at the cell edge (Figure 2G and 2H) and exhibited a predominance of filopodial extensions with some lamellipodia, rather than the sheets of lamellipodia induced by anti-NKG2D (Figure 2I). To quantify differences in the symmetry of the spreading response, the distance to the cell perimeter from the centroid of the contact with the slide was measured along 360 radii in fixed cells. The ratio of maximum to minimum lengths and the standard deviation (SD) of the radii reveal that spreading on anti-NKG2D induced symmetrical spreading (i.e., low variability in the radii), whereas cells spreading on anti-LFA-1 was asymmetrical, i.e., with a high maximum to minimum ratio and high variability in the radii (Figure 2J).

While ligation of NKG2D triggers symmetrical NK cell spreading and LFA-1 ligation results in asymmetrical spreading, both are dependent on actin remodelling, as both types of spreading were blocked when cells were treated with cytochalasin D (Figure 2K). PP2 and Ly49002, which, respectively, inhibit src-family tyrosine kinases and PI3-kinases, also blocked spreading in response to either NKG2D or LFA-1 ligation demonstrating the requirement for these specific signalling pathways.

To determine the influence of the density of activating ligands on the spreading response, slides were coated with anti-NKG2D mAb in a dilution series. Homogeneity of mAb coating at the concentrations used was confirmed by coating slides with fluorescently tagged antibodies and recording images at the slide surface (unpublished data). Over a semi-log dilution series, the proportion of cells spreading and forming the f-actin ring, as revealed by phalloidin staining, decreased with decreasing amount of mAb, with almost none responding to slides coated with 1 µg/ml mAb and ∼75% of cells responding to slides coated with 3 µg/ml (Figure 2L). Using ^125^I-labelled mAbs, the specific density of anti-NKG2D was approximately 600±230 and 3,900±1,500 molecules per µm^2^ for slides coated with 1 or 3 µg/ml respectively, and titrated linearly between these two concentrations (Figure 2L). However, among cells that formed an actin ring on slides coated with 1–3 µg/ml anti-NKG2D mAb, there was no difference in the average area to which they spread (Figure 2L). Thus, the area over which an individual cell spreads was not dose-dependent, and instead the spreading response is binary, with individual cells spreading to the full extent providing a threshold of stimulation has been reached.

### Inhibitory Receptor Signals Are Continuously Integrated during Spreading in Response to NKG2D Ligation

**Figure 3:**
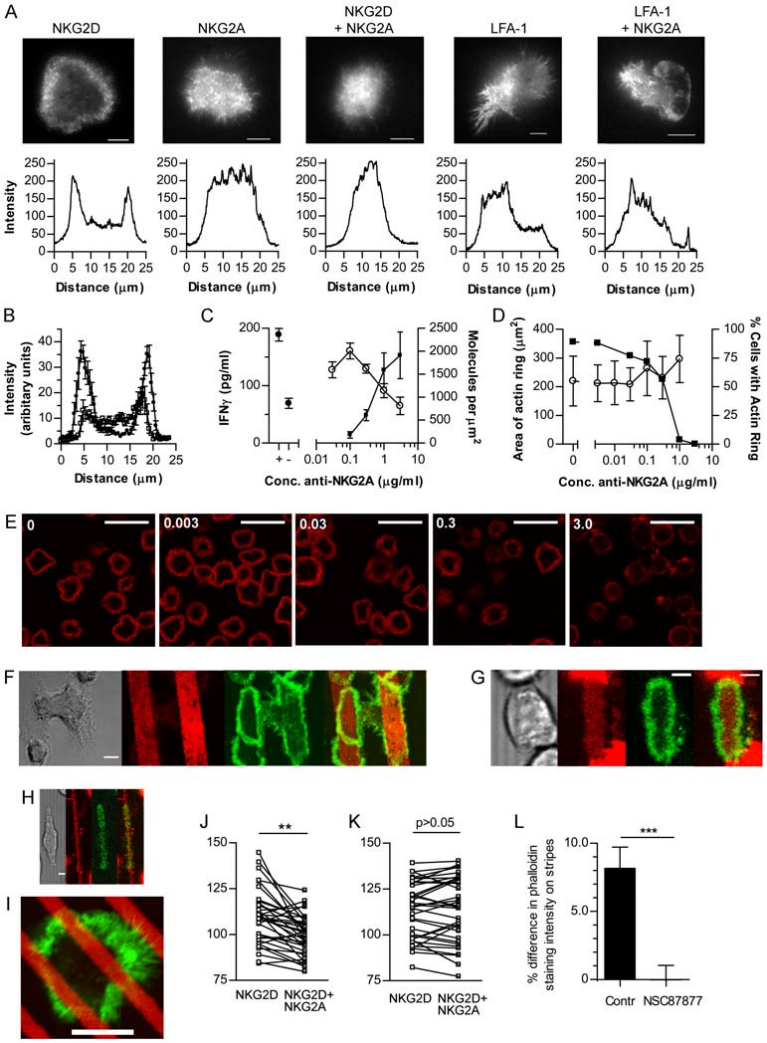
Activating and inhibitory signal integration during the spreading response. (A) TIRF microscopy of NKL YFP-actin on slides coated with anti-NKG2D, anti-NKG2A, and anti-LFA-1, or combinations thereof. Scale bar  =  10 µm. Lower panels: Fluorescence intensity through a cross section of each cell. (B) Intensity of f-actin (phalloidin-AlexaFluor633) across the diameter of NKL cells on anti-NKG2D (circles) or anti-NKG2D with anti-NKG2A (squares) (mean±SEM; *n* = 8). (C) IFNγ secretion by NKL on anti-NKG2D with anti-NKG2A (at concentrations indicated) and isotype-matched control mAb to maintain the same total concentration of Ab. For the minimal and maximal range of secretion (closed circles) the level of secretion with anti-NKG2D only (+) or isotype-matched control only (−) is shown (*n* =  3±SD). Right side shows the molecular density of anti-NKG2A on the slide (squares) and IFNγ secretion (open circles; *n* = 3±SD). (D) The proportion of NKL cells forming an actin ring (squares; *n* = 17–940) and the extent of spreading (circles; mean±SD; *n* = 36–175) on slides coated as in (C). (E) F-actin distribution (phalloidin-AlexaFluor633) in NKL cells spreading on slides coated as in (C) (concentration of NKG2A indicated). Scale bar  =  30 µm. (F) NKL on anti-NKG2D stripes interspersed with isotype-matched control mAb, showing bright field, anti-NKG2D stripes (red), and f-actin (phalloidin-AlexaFluor488; green). Scale bar  =  25 µm. (G) NKL YFP-actin (green) on anti-NKG2D (red) interspersed with mixed anti-NKG2D and anti-NKG2A. Scale bar  =  5 µm. (H) NKL YFP-actin (green) on anti-NKG2D (red) interspersed with mixed anti-NKG2D and anti-NKG2A. Scale bar  =  5 µm. (I) NKL on stripes of anti-NKG2A and anti-NKG2D (red) alternating with anti-NKG2D, showing f-actin (phalloidin-488; green). Scale bar  =  10 µm. (J) F-actin distribution in regions of cells in contact with anti-NKG2D stripes or with a mixture of anti-NKG2A and anti-NKG2D (***, *p*<0.001; *n* = 31 cells, paired *t*-test). (K) F-actin distribution following treatment with SHP inhibitor NSC87877 (*n* = 36). (L) The percent difference in f-actin accumulation between regions of cells in contact with anti-NKG2D or with anti-NKG2D and anti-NKG2A, in the presence or absence of NSC87877 (mean±SEM, *n* = 36).

We next set out to test if and how inhibitory receptor ligation affected the spreading responses caused by ligation of LFA-1 or NKG2D. Cell spreading and reorganisation of actin into a dynamic ring was visualised by total internal reflection fluorescence (TIRF) imaging of NKL transfectants expressing actin-YFP on anti-NKG2D-coated slides (Figure 3A; Video S2). Actin rapidly accumulated at the periphery of the spreading cell following contact with the slide, and this annular distribution was maintained throughout the spreading response and during contraction, after which it dissipates, as was also seen in cell–cell conjugates (Figure 1H). The actin ring was a highly dynamic structure within which waves of actin propagated, consistent with waves of membrane activity driving the symmetrical spreading process (Figure 2E). Actin ring formation did not occur on slides coated with mAb against the inhibitory receptor NKG2A (Figure 3A; Video S3). Most importantly, actin ring formation was blocked in cells exposed to a combination of anti-NKG2A and anti-NKG2D mAbs (Figure 3A and 3B; Video S4). This is consistent with previous studies showing that inhibitory receptor ligation in NK cells prevents accumulation of f-actin at NK cell synapses [14],[35],[36] and is reminiscent of how ligation of the inhibitory receptor LILRB1 inhibits TCR-mediated actin polymerization [37]. In contrast, asymmetrical spreading induced by LFA-1 ligation was unaffected by co-ligation of inhibitory receptors (Figure 3A; Videos S5 and S6).

We next titrated the coating concentration of the NKG2A mAb. Using ^125^I-labelled NKG2A mAb, in the presence of 3 µg/ml unlabelled anti-NKG2D and isotype control antibodies (to maintain the same total antibody concentration), the specific density of anti-NKG2A on the slides could be estimated, and reduced in a dose-dependent manner over the range of coating concentrations used (Figure 3C). The biological consequences of NKG2A ligation also titrated out over this range of densities, as shown by the inhibition of IFNγ production (Figure 3C). Importantly however, at increasing concentrations of anti-NKG2A mAb, the percent of cells forming an f-actin ring decreased (Figure 3D and 3E). Interestingly, the area of spreading in cells that did assemble a ring of f-actin did not alter (Figure 3D). This further demonstrates that the formation of the actin ring is a binary decision rather than occurring to an extent that is proportional to the balance of inhibitory and activating signals received.

Having shown that the integration of activating and inhibitory signals controls whether or not a cell spreads, we set out to determine whether the “decision” to spread is irreversible once spreading has been initiated. To test this, cell spreading was assessed on spatially segregated arrangements of activating and inhibitory antibodies. If NK cell spreading is determined only by the signals received at initiation of the response, cells would be expected to spread according to the ligands first encountered on contact with the slide, regardless of further mAb encountered as they spread. If signals are integrated during spreading, spreading would be expected to continue only when a cell continued to encounter activating mAb and to be stopped if the spreading cell encountered inhibitory mAb.

Microcontact printing with patterned polydimethylsiloxane (PDMS) stamps was used to coat the surface of slides with mAb patterned on a micrometer-scale. Specifically, mAb were stamped in stripes and a second mAb was applied to fill the uncoated spaces between the stamped mAb regions. The stamping method was validated by imaging the distribution of stamped and overlaid antibodies (Figure S2). When slides were stamped to display alternate stripes of anti-NKG2D and an isotype-matched control mAb, NKL cells spread in elongated shapes along the stripes of activating antibodies and the actin ring was restricted to the cell periphery along the edge of the stripe (Figure 3F). When NKL cells were stimulated by stripes of activating NKG2D antibodies interspersed with stripes containing a mixture of activating anti-NKG2D and inhibitory anti-NKG2A antibodies, spreading was again restricted to the regions of anti-NKG2D and stopped where inhibitory receptors were also ligated (Figure 3G and 3H). These data demonstrate that in regions with only activating ligands, spreading continues, but as inhibitory signals are encountered or where activating signals are absent, spreading is halted, which implies that local activating signals are needed to maintain spreading.

To test whether the actin cytoskeleton and spreading could be tightly spatially regulated in NK cells, narrower stripes (of ∼4 µm width) were used such that cells would make contact with several stripes at the same time (Figure 3I). Quantification of the abundance of f-actin in different regions of NK cells on these narrower stripes revealed that that within individual cells, the intensity of f-actin staining was significantly higher in regions interacting with activating mAb compared to regions in contact with mixed activating and inhibitory antibodies (Figure 3J). Thus, actin polymerisation is regulated according to the local balance of activating and inhibitory ligands. Blocking activity of SH2-domain containing tyrosine phosphatases SHP1 (and SHP2) involved in NKG2A-mediated inhibition, using the inhibitor NSC87877 [38], abolished the difference in f-actin intensity between regions of NKG2D and NKG2D and NKG2A engagement (Figure 3K and 3L).

Taken together with the observation of smaller synapses during inhibitory contacts, this demonstrates that if inhibitory signals are dominant, spreading and the formation of the f-actin ring are blocked. If the threshold for activation is exceeded, cell spreading continues. Rather than an irreversible commitment to spread occurring according to the signals received by the NK cell on first contact, signal integration continues during spreading and there is a constant local requirement for dominance of activating signals to continue actin polymerisation and spreading.

### LFA-1 Triggers NK Cell Migration, NKG2D Imposes a Stop Signal, and NKG2A:CD94 Delivers a Reverse-Stop Signal

**Figure 4:**
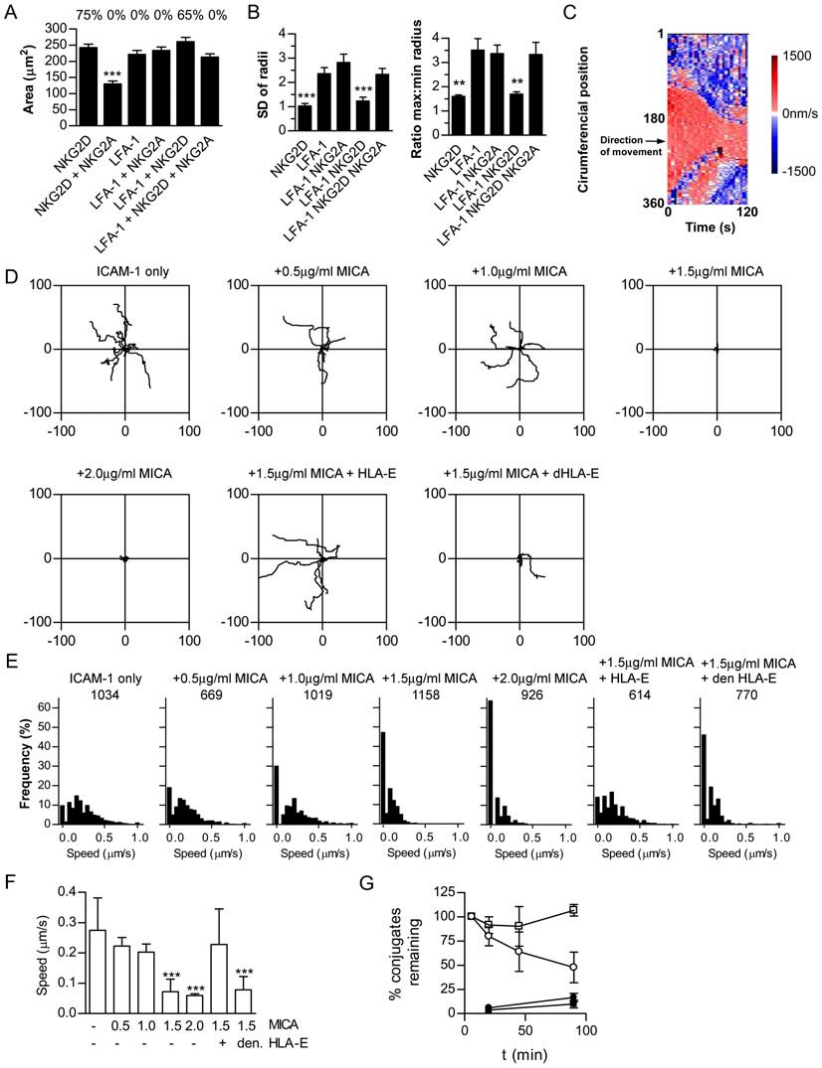
NKG2D imposes symmetry and a stop signal; NKG2A breaks symmetry and delivers a reverse-stop signal. (A) Area of contact between NKL and slides coated with combinations of LFA-1, NKG2D, and NKG2A mAb, fixed after 6 min (mean±SD, *n*  =  79–117, ***, *p*<0.0001, ANOVA). The percent of cells forming an actin ring determined by phalloidin staining is indicated above each bar. (B) Symmetry of NKL on slides coated with combinations of LFA-1, NKG2D, and NKG2A mAb. The distance from the centroid to the cell perimeter was measured at 360 equally spaced radii. Symmetry is quantified as the ratio of the longest to shortest radii and as the SD of all 360 radii (*n* = 20, ***, *p*<0.001, **, *p*<0.01 ANOVA). (C) Migration of a representative NKL cell on ICAM-1, measured by rate of radial movement of the cell circumference relative to the original cell centroid. Extension away from the original centre is coloured red and contraction blue, for each of 360 radii (*y*-axis) against time (*x*-axis). (D) NKL displacement (μm) from position at *t*  =  0, on slides coated with 2.5 µg/ml ICAM-1 in combination with MICA at concentrations indicated, with 2.5 µg/ml HLA-E or acid-denatured HLA-E (*n* = 8). (E) Frequency histograms for cell speeds measured at 10-s intervals, total number of time points analysed for each condition is indicted. (F) Average speed of cells on combinations of ICAM-1, MICA, HLA-E, or acid-denatured HLA-E (mean±SD, ***, *p*<0.001 versus ICAM-1 only, by ANOVA). Numbers indicate concentration of protein in μg/ml. (G) Duration of conjugation of YTS/KIR2DL1 with 221 (open squares) or 221/Cw6 (open circles). Cells were co-incubated in a small volume to allow conjugate formation and an excess of media added to prevent further conjugation. The proportion of NK cells remaining in conjugates was determined by flow cytometry. YTS and 221 (closed squares) and YTS-KIR2DL1 and 221-Cw6 (closed circles), which had not been premixed to form conjugates, are shown to control for conjugate formation after dilution (mean of three experiments±SD).

As NKG2D ligation and LFA-1 ligation resulted in very different NK cell morphologies we sought to understand how the signals from these receptors would combine and how they relate to cell motility. Cells stimulated on slides coated with both NKG2D and LFA-1 antibodies spread to the same extent as cells stimulated by either receptor alone, formed a ring of f-actin (Figure 4A) and spread symmetrically (Figure 4B), demonstrating that NKG2D imposes symmetrical spreading in the presence of LFA-1 ligation. Crucially, inhibitory receptor ligation broke the symmetry imposed by NKG2D in the presence of LFA-1 stimulation (Figures 4B). Thus, NKG2D imposes symmetry on NK cell spreading and this is reversed by inhibitory receptor ligation.

To study NK cell motility, slides were coated with ligands to NK cell receptors, rather than high affinity antibodies. A spreading response and formation of an f-actin ring were observed in NKL on slides coated with MICA, a natural ligand of NKG2D (unpublished data). NKL cells moved across slides coated with ICAM-1 during which cells extended leading lamellipodia in the direction of movement (Figure 4C and 4D; Video S7). T cells must receive a “stop” signal from the TCR, preventing T cell migration, in order to form a symmetrical stable synapse and become fully activated [22],[27]. Thus, to determine if NKG2D could impose a stop signal on NK cells, slides were coated with MICA in combination with ICAM-1. Coating slides with 2.5 µg/ml ICAM-1 in the presence of MICA generated an ICAM-1 density on the slide surface of 2,400±440 molecules per µm^2^ (mean of four wells±SD), as determined using ^125^I-labelled protein. MICA was used at 2.0 µg/ml (leading to densities of protein on the slide of 4,500±1,500 molecules per µm^2^), 1.5 µg/ml (2100±1,100), 1.0 µg/ml (1,600±120) and 0.5 µg/ml (545±85). MICA imposed symmetrical spreading and delivered a stop signal (Video S8). At increasing densities of MICA the average speed of cell migration was reduced (Figure 4F). Analysis of cell speeds in each 10-s time frame, revealed that increasing concentrations of MICA increased the proportion of time cells spend stopped (Figure 4E).

Previously, studies with a rat NK cell line showed that cell motility was preserved during inhibitory interactions with target cells [39]. Also, there is evidence that a reduction in conjugate formation occurs at inhibitory synapses, but an earlier study showed that blocking inhibitory class I MHC proteins did not change the extent of NK cell conjugation [40],[41]. Thus we next set out to clarify whether inhibitory receptors act to deliver a “reverse-stop” signal that restores cell motility and decreases the duration of the synapse. HLA-E was coated onto slides at 2.5 µg/ml, which produced a density of 11,700±2,700 molecules/µm^2^. Crucially, the stop signal delivered by MICA was reversed when slides were coated with ICAM-1, MICA, and the NKG2A:CD94 ligand, HLA-E, and not when HLA-E had been denatured by acid (Figure 4D–4F).

To next test whether inhibitory receptor ligation causes a reduction in the duration of conjugation, fluorescently labelled YTS/KIR2DL1 were co-incubated with target 221 or 221/Cw6 in a small volume to allow conjugate formation and an excess of media added to dilute the cells and thus prevent further cell–cell conjugation. The proportion of NK cells in conjugates after different times was determined by flow cytometry. This revealed that the presence of inhibitory receptor ligands dramatically reduced the time of engagement between NK cells and target cells (Figure 4G). Thus, inhibitory receptor ligation can prevent cytotoxicity by blocking formation of a large stable symmetrical synapse and instead favouring a migratory configuration, which reduces the duration of intercellular conjugation.

## Discussion

In summary, we have demonstrated that the balance of activating and inhibitory ligands is continually locally assessed by NK cells following contact with a target cell. Under physiological conditions, NK cell activation results from integration of many activating and inhibitory signals from surface receptors interacting within immune synapses. Here we have studied the outcome of cross-talk between the integrin LFA-1, the activating receptor NKG2D, and the inhibitory receptor NKG2A. If activating signals dominate, the NK cell stops and spreads lamellipodia across the target to form a large stable synapse, simultaneously forming an actin ring and polarising towards the target cell. Importantly, inhibitory receptor signalling can break synapse symmetry and reverse the stop signal, allowing cells to migrate and resulting in a reduced dwell time on target cells. This establishes a specific framework by which the balance of activating and inhibitory signals is translated into an inhibitory or cytolytic response.

In cytotoxic T lymphocytes, the formation of a peripheral actin ring polarises the MTOC and directs cytotoxic granules to the target [42]. Using photoactivatable peptides, it has been shown that MTOC polarisation is induced within 2 min of TCR engagement, and therefore occurs concurrently with T cell spreading [43]. Similarly, spreading and actin ring formation in NK cells also occurred concurrently with granule polarisation. T cell spreading is mediated by recruitment of adaptor proteins such as nck and Wiscott-Aldrich Syndrome protein (WASp), which direct formation of the f-actin-rich ring at the edge of the synapse [44],[45]. WAS patients are impaired in both T cell- and NK cell-mediated cytotoxicity [46],[47] consistent with cytolytic T cells and NK cells using similar processes in spreading and the delivery of cytolytic granules.

For B cells the extent of spreading is proportional to the affinity of the B cell receptor for antigen and the density of antigen [48]. As the B cell spreads it captures further antigen, which is processed and presented to T cells. Thus, spreading provides a structural basis for B cell affinity discrimination. In contrast, NK cell receptors are germ line encoded and thus do not exhibit the log scale variation in affinity exhibited by B cell and T cell receptors. Instead, NK cells must determine the relative density of activating and inhibitory ligands [10],[49]. Here, we found that the density of activating and inhibitory ligands determines whether or not an NK cell undergoes a spreading response, and that the extent of spreading is not proportional to the balance of signalling but rather occurs if a threshold of activation is surpassed. Thus the spreading response in the NK cell is binary and this reflects a necessity for the outcome of the interaction of an NK cell with a target cell to be all-or-nothing, at least in the case of a cytolytic response, i.e., either killing or sparing the target cell. In T cells, competing positive and negative feedback pathways involving ERK and SHP-1 lead to an all-or-none biological response to TCR ligation [50],[51], and analogous pathways may lead to binary spreading responses in NK cells.

Microcontact printing of spatially segregated activating and inhibitory mAb was employed to demonstrate that spreading and actin polymerisation stop when a spreading cell does not encounter a dominance of activating ligands. Thus signal integration continues during the spreading response and the continued dominance of local activating signals is necessary for spreading to continue. Functionally, this continuous signal integration during spreading is likely to be crucial in preventing misdirection of a cytolytic response towards normal healthy neighbouring cells within crowded tissue, ensuring that the activating synapse forms only towards an appropriate target cell.

The T cell synapse has recently been described as an adhesive junction between cells exhibiting radial symmetry, whereas a moving adhesive junction, with a leading lamellipodium, has been termed a “kinapse” [27],[31]. It remains to be established whether analogous moving adhesive contacts made by NK cells are capable of triggering a response such as secretion of particular cytokines, as found for T cells. There is clearly a great deal of similarity between NK cells and cytotoxic T lymphocytes, the latter also found to migrate on ICAM-1 monolayers where co-ligation of NKG2D favoured formation of an actin ring [52]. Ligation of the inhibitory receptor CTLA-4 provides a “reverse-stop” signal to T cells and thus reduces the duration of T cell contact with an APC, which may be a key mechanism by which it inhibits T cell activation [53],[54]. Taken together with our data, it emerges that inhibitory receptor signals can act, for both T cells and NK cells, as regulators of conjugation, by breaking the symmetrical spreading imposed by activating receptors and reducing the time of contact between cells.

## Materials and Methods

### Cell Lines

NKL were maintained in 10% FCS, 100 µg/ml streptomycin, 100 U/ml penicillin, L-glutamine, β-mercaptoethanol, nonessential amino acids in RPMI-1640 (all Invitrogen; complete medium) with 100 U/ml IL-2. NKL cells were transfected with pEYFP-actin or pEYFP-mem vectors (Clontech) by electroporation (Amaxa Biosystems) and stable transfectants selected using 1 mg/ml G418. YTS and YTS/KIR2DL1 and the EBV transformed B cell line 721.221 (221) and 221 transfected to express HLA-Cw6 (221-Cw6) or MICA-YFP were obtained and cultured as described [55]–[57]. Primary human NK cells were isolated from healthy donor buffy coats under negative magnetic selection (Miltenyi) and cultured as described [58].

### Imaging and Analysis of NK: Target Cell Conjugates

NK and target cells were mixed in chamber slides and live cell–cell conjugates were imaged at 5% CO_2_, 37°C by confocal microscopy (Leica SP5 RS) using a 63× water immersion lens (NA 1.2). To measure NK cell polarisation, intracellular membranes, stained with DiD (Invitrogen), were mapped as a 3D object using software (Volocity, Improvision). Polarisation was expressed as the distance of the centroid of this object to the centre of the synapse as a proportion of the cell length, measured from the centre of the synapse to the other cell pole.

### Preparation of Coated Slides

Protocols for preparing chamber slides (Nunc) were adapted from published protocols [59]. Briefly, slides were cleaned using acidified 70% ethanol, coated with 0.01% poly L-lysine, then mAb or recombinant protein (at 10 µg/ml PBS unless indicated otherwise), then blocked with complete medium. Antibodies to NKG2D (Clone 149810, R&D systems), NKG2A (clone 131411, R&D systems), LFA-1 (Clone G43-25B, Pharmingen), pan-MHC class I (Clone W6/32), or murine IgG isotype controls (Pharmingen), recombinant ICAM-1, or ICAM-1-Fc and MICA-Fc chimeras (R&D systems) were used. HLA-E monomers were produced and refolded with the HLA-G peptide VMAPRTLFL, as described [11]. HLA-E was denatured by mixing with an equal volume of citrate/phosphate buffer (0.131 M citric acid, 0.066 M Na_2_HPO_4_ [pH 3.3]) for 1 min [60].

To determine the density of molecules coated on slides, proteins were radiolabelled with ^125^I (ICN Chemicals) using iodogen coated tubes according to manufacturer's instructions (Pierce Chemicals) and dialysed overnight against PBS. Slides were coated with ligands or antibodies under the conditions described above, in combination with unlabelled proteins as appropriate, in quadruplicate wells. Slides were dismantled and the amount of protein bound per well was counted using a gamma counter (Hewlett Packard). The coating density was calculated (molecules per µm^2^) using reference radiolabelled samples of known concentrations and molecular weight of each protein as 150, 79, 100, and 48 kDa for antibodies, ICAM-Fc, MICA-Fc, and HLA-E, respectively.

Microcontact printing was performed using PDMS stamps in an adaptation of published procedures (Sylgard 184 Silicone Elastomere kit, Dow Corning) [61]. Stamps were coated for 1 h with 100 µg/ml PBS mAb mixed with 15 µg/ml AlexaFluor 635 anti-rat IgG (Invitrogen) to allow visualization of antibodies, then washed with ddH_2_0, air dried, and pressed onto poly L-lysine coated slides [62]. Stripes were overlaid with of 5 µg/ml mAb in PBS for 1 h then blocked with 1% bovine serum albumin in PBS.

### Imaging NK Cell Spreading and Migration on Coated Slides

Cells were added to coated chamber slides in 25 mM HEPES in complete medium. In some cases cells were preincubated for 30 min with PP2, LY294002, cytochalasin D, or the SHP1/2 inhibitor NSC87877 [38] at the concentrations indicated. Bright field, fluorescence, and IRM images were captured at the slide surface every 5 or 10 s for up to 30 min using an inverted confocal microscope (described above) with either 63× or 20× lens (NA 0.7). Cell speeds and spreading were quantified (Volocity, Improvision). For TIRF microscopy, NKL-YFP actin cells were added to coated chamber slides. Single colour fluorescent images were obtained on an inverted microscope (Axiovert 200, Carl Zeiss) modified for objective-type TIRF (Till Photonics), using a 100× objective (NA 1.45, Carl Zeiss) and recorded using a charge-coupled device camera (PCO SensiCam) and software (Image J).

To visualise f-actin, cells were fixed (Cytofix/cytoperm, BD Pharmingen) 6 min after addition to coated chamber slides, washed with 0.05% Tween/PBS and stained with 2 U/ml phalloidin-AlexaFluor 635 or phalloidin-AlexaFluor 488 (Invitrogen) in 5% horse serum/3% BSA in buffer containing detergent (Permwash, Pharmingen). The presence of an actin ring was defined as a broad ring of intense f-actin staining (revealed by phalloidin) surrounding an unstained cell centre. Filopodia were defined morphologically as long thin actin-rich extensions, whereas lamellipodia were defined as broad membranous sheets extending from the cell body. Each cell was then scored for the presence of filopodia, lamellipodia, both, or neither, in a single confocal image taken at the slide surface. To measure the area of spreading, the edge of the cell was defined by fluorescence intensity (using Volocity software), and it was confirmed that this correlated with the edge of the cell identified in the reflected (IRM) image. To quantify the intensity of phalloidin staining on micropatterned mAb, binary masks were produced, of the fluorescent stripes and the overlay, which were multiplied by the channel containing phalloidin staining, enabling quantification of f-actin on each surface (using Image J software).

Cell symmetry and the directional rate of extension or contraction, was determined similarly to published analysis [27], using a custom programme written in G (Labview software). The perimeter of the cell was defined according to fluorescence intensity thresholding and the centroid located. The distance was measured from the centroid to the perimeter along 360 evenly spaced radii. The SD of these distances and ratio of maximum to minimum distance were used to determine cell symmetry. To measure radial speed of spreading and contraction, this programme determined the rate of change of the radius at each angle, between frames.

### Conjugation Assay

YTS/KIR2DL1 stained with DiD and 221 cells, with or without expression of HLA-Cw6 stained with DiO, were incubated together at 10^6^/ml in 100 µl, for 20 min to allow conjugate formation, then diluted to stop conjugate formation, in 10 ml complete medium in petri dishes. Conjugates were fixed with 4% paraformaldehyde at 6, 20, 45, and 90 min after dilution, and the proportion of single and double positive fluorescent events (conjugates) analysed by flow cytometry (FACSCalibur, BD Biosciences) and FlowJo software.

### IFNγ Secretion Assay

Chamber slides were prepared as above and coated with 3 µg/ml NKG2D, mixed with NKG2A mAb, at a range of concentrations, and isotype control antibody, to a final total concentration of 6 µg/ml Ab in PBS at 4°C overnight. After blocking with complete medium, 1.5 × 10^5^ NKL were added per well in 250 µl complete medium. Alternatively, 1.5 × 10^5^ NKL were mixed with 1.5 × 10^5^ target cells (221, 221-MICA, or 221 HLA-E) in a total volume of 250 µl complete medium. Cells were incubated for 24 h at 37°C, 5% CO_2_, after which the supernatant was assayed for IFNγ by ELISA according to manufacturer's instructions (matched anti-human IFNγ antibody pair; R&D systems).

### Statistical Analysis

Data were analysed by *t*-test or one-way ANOVA as indicated.

## Supporting Information

Figure S1 **Inhibition of IFN**γ **production by target cell expression of HLA-E.** To confirm that 221 transfected to express HLA-E were inhibitory in our hands, we incubated 1.5 × 10^5^ NKL cells with 1.5 × 10^5^ target cells (221, 221-MICA, or 221-HLA-E) in total volume of 250 µl in triplicate, for 24 h. Supernatants were then assayed by ELISA for IFNγ production (shown as mean +SD).
(0.64 MB TIF)

Figure S2 **The homogeneity and density of stripes achieved by microcontact printing.** To confirm the efficacy of our microcontact printing procedure, 100 µg/ml NKG2D mAb was mixed with 15 µg/ml anti-rat IgG-AlexaFluor 635 to identify regions of the stamped mAb, and applied to a lysine coated glass slide using a PDMS stamp. The slide was overlaid with anti-NKG2D mAb at 5 µg/ml and total mAb detected with anti-mouse AlexaFluor-488. Image shows the distribution of the stamped mAb (red) overlaid with total mAb (green); scale  =  10 µm. The graph shows the intensity profile (indicated by a line on the image) for stamped (red) and total (green) mAb and indicates that the distribution of the stamped mAb is restricted to the stripes and that the overall density of mAb in the stripes and regions of overlay are the same.
(2.93 MB TIF)

Video S1 **NKG2D ligation causes spreading in NKL cells.** NKL cells were labelled with the membrane dye DiD and were stimulated by glass surfaces coated in NKG2D mAb and imaged every 5 s at the slide surface by confocal microscopy.
(8.11 MB MOV)

Video S2 **NKL cell transfected with YFP-actin stimulated on a glass slide coated in NKG2D mAb only.** Images were recorded using TIRF microscopy. Scale  =  5 µm. Speed  =  15×.
(1.07 MB MOV)

Video S3 **NKL cell transfected with YFP-actin stimulated on a glass slide coated in NKG2A mAb.** Images were recorded using TIRF microscopy. Scale  =  5 µm. Speed  =  15×.
(9.48 MB MOV)

Video S4 **NKL cell transfected with YFP-actin stimulated on a glass slide coated in an equal mixture of NKG2D and NKG2A mAb.** Images were recorded using TIRF microscopy. Scale  =  5 µm. Speed  =  15×.
(2.36 MB MOV)

Video S5 **NKL cell transfected with YFP-actin stimulated on a glass slide coated in LFA-1 mAb.** Images were recorded using TIRF microscopy. Scale  =  5 µm. Speed  =  15×.
(5.58 MB MOV)

Video S6 **NKL cell transfected with YFP-actin stimulated on a glass slide coated in and equal mixture of LFA-1 and NKG2A mAbs.** Images were recorded using TIRF microscopy. Scale  =  5 µm. Speed  =  15×.
(7.63 MB MOV)

Video S7 **NK cells migrate on ICAM-1.** NKL cells were transfected to express YFP-mem and were stimulated by glass slides coated with ICAM-1. Bright field, fluorescent, and IRM images were recorded every 10 s. Field of view  =  246 × 246 µm.
(4.25 MB MOV)

Video S8 **NK cells are stopped by MICA.** NKL cells were transfected to express YFP-mem and were stimulated by glass slides coated with ICAM-1 and MICA. Bright field, fluorescent and IRM images were recorded every 10 s. Field of view  =  246 × 246 µm.
(4.47 MB MOV)

## Abbreviations

IFNγ: interferon gamma
IRM: interference reflection microscopy
KIR: killer-cell immunoglobulin-like receptor
LILR: leukocyte Ig-like receptor
MTOC: microtubule organizing centre
NK cell: natural killer cell
PDMS: polydimethylsiloxane
SD: standard deviation
TIRF: total internal reflection fluorescence
YFP: yellow fluorescent protein
