# Supplementary figures and images for: Natural Killer Cell Signal Integration Balances Synapse Symmetry and Migration
Source: PLoS Biol. 2009 Jul 28;7(7):e1000159. doi: 10.1371/journal.pbio.1000159 (PMC2707003; doi:10.1371/journal.pbio.1000159)

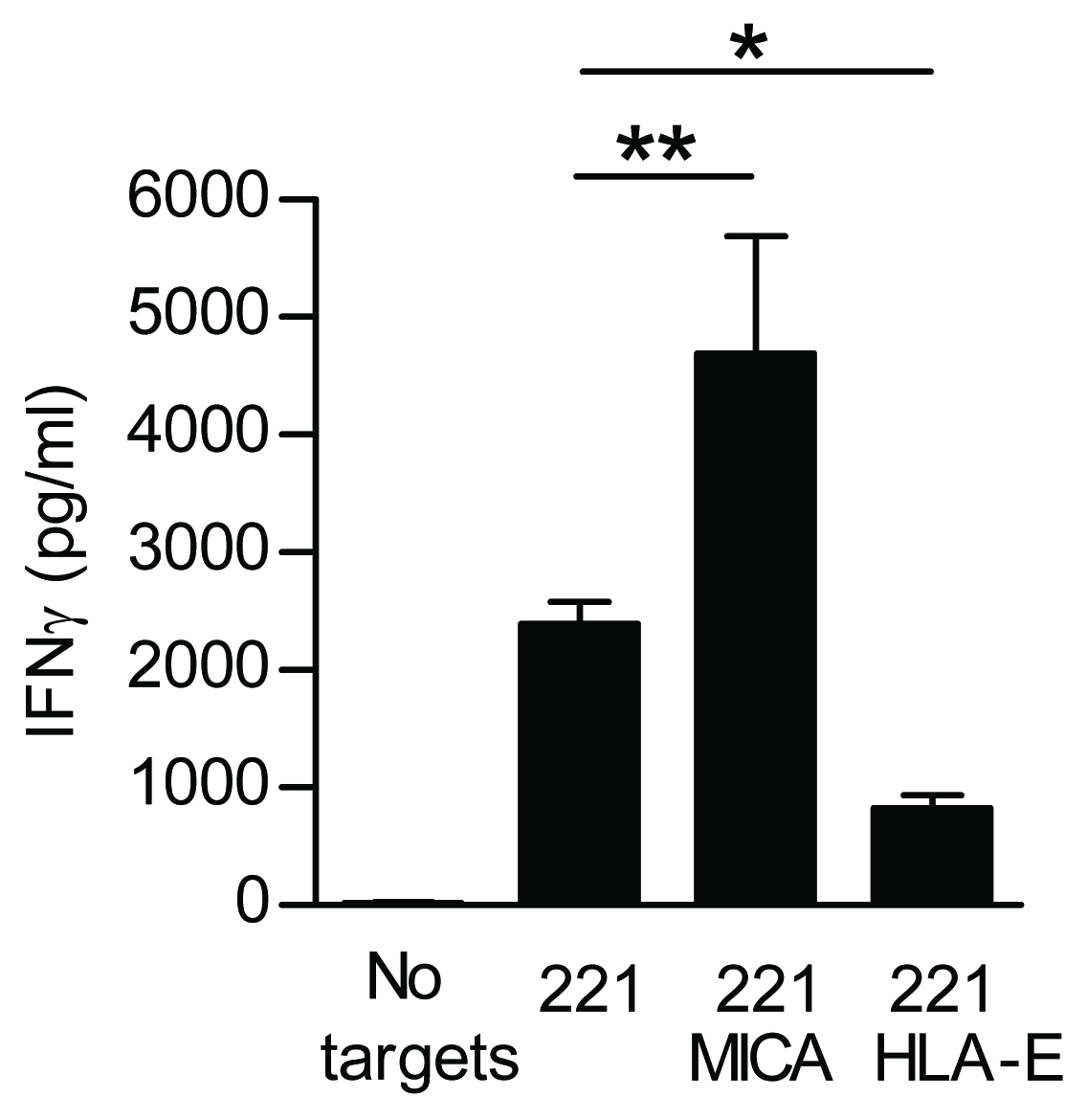

Supplement: Figure S1 — Inhibition of IFNγ production by target cell expression of HLA-E. To confirm that 221 transfected to express HLA-E were inhibitory in our hands, we incubated 1.5 × 10^5^ NKL cells with 1.5 × 10^5^ target cells (221, 221-MICA, or 221-HLA-E) in total volume of 250 µl in triplicate, for 24 h. Supernatants were then assayed by ELISA for IFNγ production (shown as mean +SD). (0.64 MB TIF) [file pbio.1000159.s001.tif]

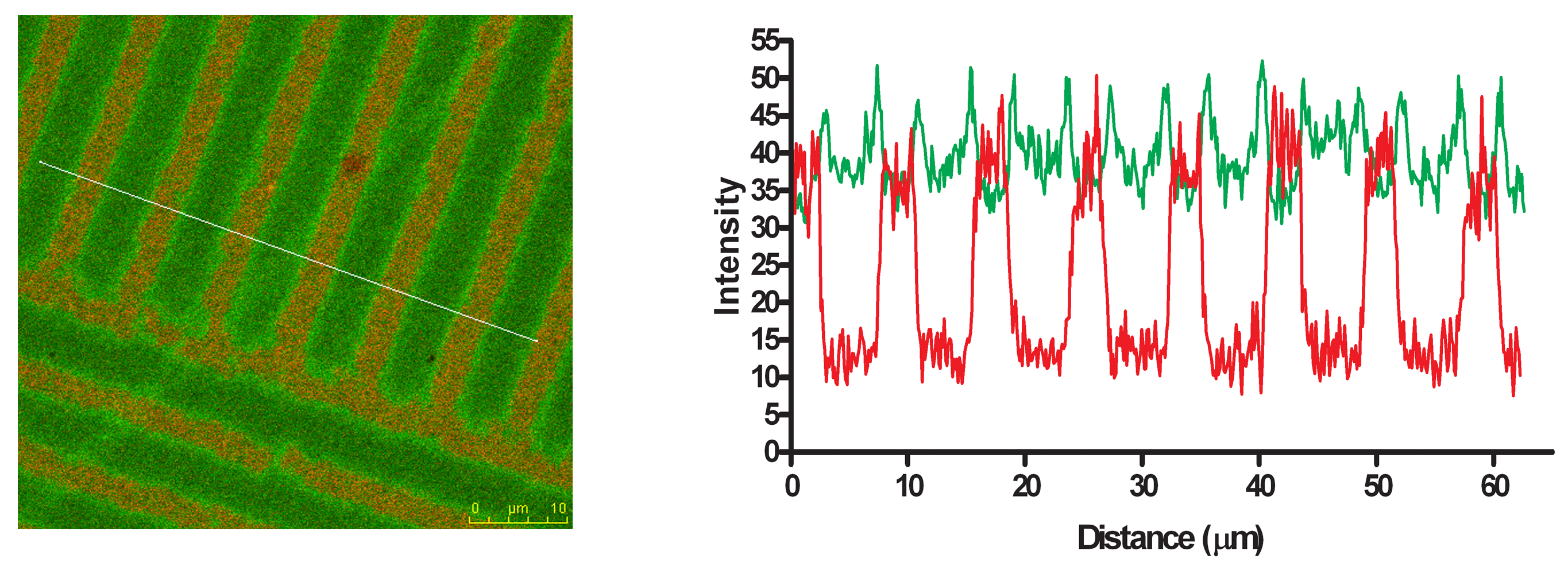

Supplement: Figure S2 — The homogeneity and density of stripes achieved by microcontact printing. To confirm the efficacy of our microcontact printing procedure, 100 µg/ml NKG2D mAb was mixed with 15 µg/ml anti-rat IgG-AlexaFluor 635 to identify regions of the stamped mAb, and applied to a lysine coated glass slide using a PDMS stamp. The slide was overlaid with anti-NKG2D mAb at 5 µg/ml and total mAb detected with anti-mouse AlexaFluor-488. Image shows the distribution of the stamped mAb (red) overlaid with total mAb (green); scale = 10 µm. The graph shows the intensity profile (indicated by a line on the image) for stamped (red) and total (green) mAb and indicates that the distribution of the stamped mAb is restricted to the stripes and that the overall density of mAb in the stripes and regions of overlay are the same. (2.93 MB TIF) [file pbio.1000159.s002.tif]
